# The Peripheral Myeloid Expansion Driven by Murine Cancer Progression Is Reversed by Radiation Therapy of the Tumor

**DOI:** 10.1371/journal.pone.0069527

**Published:** 2013-07-25

**Authors:** Marka R. Crittenden, Talicia Savage, Benjamin Cottam, Keith S. Bahjat, William L. Redmond, Shelly Bambina, Melissa Kasiewicz, Pippa Newell, Andrew M. Jackson, Michael J. Gough

**Affiliations:** 1 Earle A. Chiles Research Institute, Robert W. Franz Cancer Center, Providence Portland Medical Center, Portland, Oregon, United States of America; 2 The Oregon Clinic, Portland, Oregon, United States of America; 3 Providence Hepatobiliary and Pancreatic Cancer Program, Providence Portland Medical Center, Portland, Oregon, United States of America; 4 Tumour Interactions Group, Academic Unit of Clinical Oncology, University of Nottingham, Nottingham, United Kingdom; University of Michigan School of Medicine, United States of America

## Abstract

Expansion of myeloid-lineage leukocytes in tumor-bearing mice has been proposed as a cause of systemic immunosuppression. We demonstrate that radiation therapy of tumors leads to a decline in myeloid cell numbers in the blood and a decrease in spleen size. The frequency of myeloid cells does not decline to the level seen in tumor-free mice: we demonstrate that metastatic disease can prevent myeloid cell numbers from returning to baseline, and that tumor recurrence from residual disease correlates with re-expansion of myeloid lineage cells. Radiation therapy results in increased proliferation of T cells in the spleen and while T cell responses to foreign antigens are not altered by tumor burden or myeloid cell expansion, responses to tumor-associated antigens are increased after radiation therapy. These data demonstrate that myeloid cell numbers are directly linked to primary tumor burden, that this population contracts following radiation therapy, and that radiation therapy may open a therapeutic window for immunotherapy of residual disease.

## Introduction

Myeloid cells have an important role in the development and progression of cancer. Tumor-associated macrophages are critical for angiogenesis, invasion, metastasis, immunosuppression and response to therapy [1], [2], [3]. Recently studies have focused on the population of myeloid cells that is frequently expanded in the peripheral blood of cancer patients [4], [5]. Certain mouse models are associated with extreme myeloid expansions detectible in the tumor, spleen and peripheral blood, and these myeloid cells are able to suppress T cell activation *in vitro* [6], [7], [8].

Transplantable tumor models with their clonally identical cancer cells provide a useful model to study the key features of myeloid expansion. If the myeloid expansion is linked to the number of cancer cells, then treatment of the primary tumor should prevent myeloid expansion. Gemcitabine and 5-FU chemotherapy have been shown to control the myeloid expansion in the spleens of tumor-bearing mice [9], [10], [11]. However, each of these agents has been described to have a direct inhibitory effect on myeloid populations *in vitro* [10], [11]. While these are not particularly myelotoxic chemotherapies, the potential systemic effects of chemotherapies on actively proliferating myeloid precursors can confound the contribution of reduced tumor burden. Surgical removal of the primary tumor also causes a decrease in myeloid cells [9], [12]. It is interesting that this effect is incomplete, as cells do not return to naïve levels. These data suggest that tumors have an effect on myeloid cells that persists beyond excision. However, in this model the effect of tumor excision is transient, as myeloid expansion returns with recurrence of the primary tumor and metastases [12]. Therefore, these data could be reinterpreted to suggest that residual cancer cells may prevent a full normalization of myeloid numbers. In the surgical model, trauma may also act as a confounding factor. Trauma has been shown to cause a mobilization of myeloid cells with similar phenotypic, morphologic and function properties to tumor-induced myeloid cells [13]. This trauma-induced myeloid expansion may conceal the extent of the reduction in myeloid cells caused by surgical removal of the primary tumor, and add to any myeloid expansion sustained by residual disease.

The consequence of tumor radiation therapy to systemic myeloid populations has not been described. Radiation therapy can be delivered in a highly site-specific manner, resulting in control of targeted tumors and under normal circumstances there is no effect on tumors outside the target field. Thus, radiation therapy provides a technique to affect of the primary tumor on peripheral myeloid cells without the confounding effects of chemotherapy and surgery. We demonstrate that radiation therapy of 4T1 tumors causes a decline in myeloid cell numbers in the blood and a decrease in spleen size. Systemic disease, as measured by lung metastases, is not affected by radiation therapy of the primary tumor. We demonstrate that myeloid expansion closely follows primary tumor growth, but that following treatment, myeloid numbers do not decline to the level seen in tumor-free mice, suggesting that following local treatment, residual local and metastatic disease combine to sustain myeloid numbers. When primary tumors recurred as a result of residual local disease, myeloid expansion returned. We demonstrate that while myeloid numbers are regulated by tumor growth and influenced by radiation therapy, T cell numbers do not change. However, radiation therapy of the primary tumor improved the myeloid: CD8 ratio and results in increased proliferation of endogenous T cells in the spleen. To test whether myeloid expansion and contraction affected in vivo T cell responses, we measured the antigen specific response to vaccine-associated and tumor-associated antigens. We demonstrated that there was no change in the *in vivo* response to *Listeria monocytogenes* vaccination in tumor-bearing and treated mice but that the combination of radiation therapy with vaccination results in increased responses to vaccine antigen shared with the tumor. These data support the hypothesis that myeloid expansion is directly linked to tumor burden, that these cells contract following radiation therapy, and that radiation therapy may open a therapeutic window for immunotherapy of residual disease.

## Materials and Methods

### Ethics

All animal protocols were approved by the Earle A. Chiles Research Institute IACUC (Animal Welfare Assurance No. A3913-01).

### Animals and Cell Lines

The 4T1 mammary carcinoma cell line [14] (BALB/c) was obtained from the ATCC (Manassas, VA). The Panc02 murine pancreatic adenocarcinoma cell line [15] (C57BL/6) was kindly provided by Dr Woo (Mount Sinai School of Medicine, NY). 6–8 week old C57BL/6 mice and BALB/c were obtained from Charles River Laboratories (Wilmington, MA) for use in these experiments.

### Antibodies and Reagents

Fluorescently-conjugated antibodies CD11b-AF700, Gr1-PE-Cy7, IA (MHC class II)-e780, Ly6C-PerCP-Cy5.5, CD4-e450, FoxP3-e450, CD25-APC, CD4-PerCP Cy5.5, CD8-FITC, IFNγ-APC, and CD40L-PE were purchased from Ebioscience (San Diego, CA). Ki67-FITC, CD4-v500, TNFα-PE-Cy7 and Ly6G-FITC were purchased from BD Biosciences (San Jose, CA). CD8-PE-TxRD was purchased from Invitrogen (Carlsbad, CA).

### Radiation Therapy of Tumors

Tumors were inoculated s.c. in the right leg below the knee at a dose of 5×10^4^ 4T1 cells or 2×10^5^ Panc02 and allowed to establish for 14 days before initiation of treatment. Dosing was based on recent clinical studies [16], with three daily 20 Gy treatment fractions given using an Elekta Synergy linear accelerator (Atlanta, GA) with 6 MV photons incorporating a half beam block to minimize dose to the torso and 1 cm bolus.

### Clonogenic Analysis of Metastatic Cancer Cells

For clonogenic analysis of metastatic cancer cells, the lungs were dissected into approximately 2 mm fragments followed by agitation in 1 mg/mL collagenase (Invitrogen), 100 µg/mL hyaluronidase (Sigma, St Louis, MO), and 20 mg/mL DNase (Sigma) in PBS for 1 hr at room temperature. The digest was filtered through 100 µm nylon mesh to remove macroscopic debris. Serial dilutions of tumor cells were seeded to 6-well tissue culture plates in media containing 60 µM 6-thioguanine to select for cancer cells over stromal cells and colonies were counted after 7 days. The serial dilution and the colony count were used to calculate the number of clonogenic cancer cells in the original organ.

### Flow Cytometry of Myeloid Cells in the Blood and Spleen

The expansion of myeloid cells in the peripheral blood was measured using a whole blood bead assay. Whole blood was harvested into EDTA tubes from live mice via the saphenous vein, and 25 µl of fresh blood was stained directly with fluorescent antibody cocktails. A known number of AccuCheck fluorescent beads (Invitrogen) were added to each sample, then red blood cells were lysed with Cal-Lyse whole blood lysing solution (Invitrogen), and samples analyzed on a BD LSRII flow cytometer. We determined the absolute number of cells in the sample based on comparing cellular events to bead events (cells/µl). For flow cytometry analysis of splenocytes, homogenized spleens were washed and stained with antibodies specific for surface antigens, then cells were washed and fixed using a T regulatory cell staining kit (EBiosciences) and intracellularly stained for FoxP3 and Ki67. The proportion of each infiltrating cell type was analyzed on a BD LSRII. Flow sorting of blood cells was performed using a BD FACSAria Cell Sorter to greater than 98% purity. The morphology of the sorted cell populations was determined by cytospin followed by DiffQuick staining. Blood smears were stained using Wright’s-Giemsa stain (Ricca Chemical Company, Arlington, TX). Images were acquired using a Nikon Eclipse TE2000-S fluorescence microscope with NIS-Elements acquisition and analysis software, or on a Leica SCN400 slide scanner.

### Cytokine Bead Assay

Tumors were harvested on ice and homogenized in 4.5 µl PBS containing HALT protease inhibitor per mg tissue. The cell debris was removed by centrifugation at 14000 g for 15 minutes at 4°C, and supernatants were stored in aliquots at −80°C until used. Cytokine levels in the supernatants were detected using a murine multiplex bead assay (Life Technologies, Grand Island, NY) and read on a Luminex 100 array reader. Cytokine concentrations for replicates of each tumor sample were calculated according to a standard curve.

### Bacterial Strain and Vaccination

ActA-deleted (Δ*actA*) *L. monocytogenes* expressing the AH1-A5 peptide Lm-AH1-A5) have been previously described [17]. Bacteria were grown to midlog in brain-heart infusion broth, washed and resuspended in dPBS for injection. A dose of 5×10^6^ CFU was delivered intravenously and confirmed by plating of residual inoculum. Control mice or mice bearing 4T1 tumors left untreated or treated as above with three daily 20 Gy treatment fractions were vaccinated 1 day following the final radiation dose with 5×10^6^ CFU Lm-AH1-A5. Spleens were harvested 7 days following vaccination and cell suspensions were stimulated with 2 µM of LLO_91–99_ (GYKDGNEYI), AH1 (SPSYVYHQF), or DMSO vehicle in the presence of brefeldin A for 5 hours at 37°C. Stimulated cells were washed and stained with CD4-PerCP Cy5.5 and CD8-FITC, then fixed and permeabilized using a BD Cytofix/Cytoperm plus kit (BD Biosciences) and frozen at −80°C. For analysis cells were thawed and intracellularly stained with IFNγ-APC, TNFα-PE-Cy7 and CD40L-PE. Cells were washed and analyzed on a BD LSRII Flow Cytometer and the data was interrogated using BD FACSDiva (BD Biosciences) and FloJo (Tree Star, Ashland, OR).

### Statistics

Data were analyzed and graphed using Prism (GraphPad Software, La Jolla, CA). Blood myeloid numbers over time were fitted to second order polynomial curves using Prism. Individual data sets were compared using Student’s T-test and analysis across multiple groups was performed using ANOVA with individual groups assessed using Tukey’s comparison.

## Results

**Figure 1:**
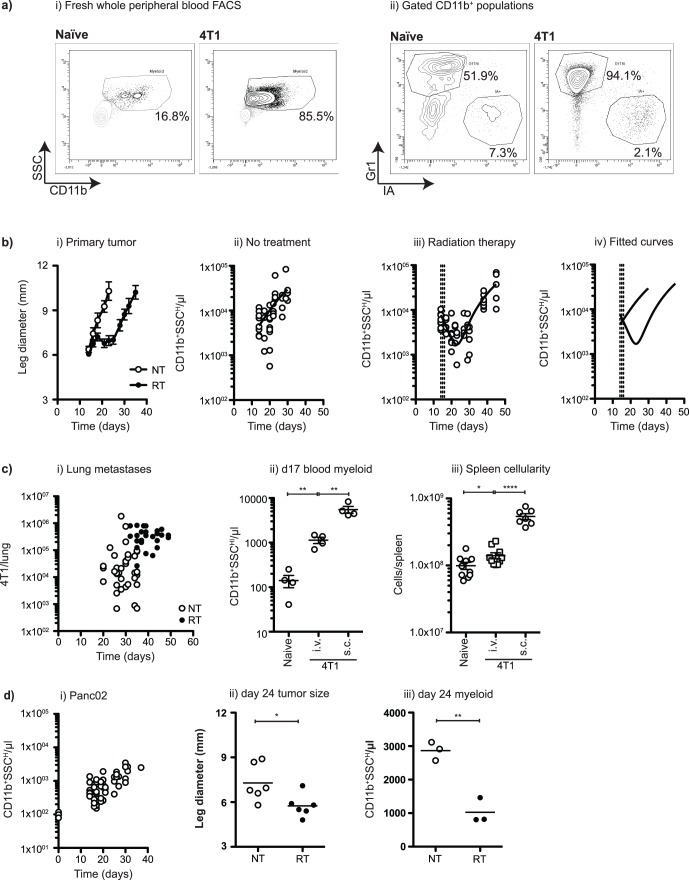
Tumor radiation results in a myeloid contraction. a) i) Flow cytometry of fresh whole peripheral blood from naïve and 4T1 tumor-bearing BALB/c mice, showing CD11b^+^SSC^hi^ myeloid populations. ii) Gr1 and IA (MHC class II) staining on gated CD11b^+^SSC^hi^ myeloid populations from naïve and 4T1 tumor-bearing mice. b) i) Mean and standard error of leg diameter of BALB/c mice bearing 4T1 tumors left untreated (NT) or treated beginning on day 14 with 3 daily doses of 20 Gy focal radiation (RT). Myeloid cells/µl peripheral blood of ii) mice left untreated and iii) mice treated beginning on day 14 with 3 daily doses of 20 Gy focal radiation. iv) fitted curves from graphs ii) and iii) plotted without measurements. c) i) Clonogenic assays of lung metastases present at euthanasia in BALB/c mice bearing 4T1 tumors left untreated (empty circles – NT) or treated beginning on day 14 with 3 daily doses of 20 Gy focal radiation (filled circles – RT). ii) Myeloid cells/µl peripheral blood of naïve mice (Naïve) and mice day 17 following injection of 4T1 i.v. (i.v.) or s.c. (s.c.) iii) Total spleen cellularity in naïve mice (NT) and mice day 24 following injection of 4T1 i.v. (i.v.) or s.c. (s.c.) d) i) Myeloid cells/µl peripheral blood of C57BL/6 mice bearing Panc02 tumor harvested at different time points. ii) day 24 leg diameter and iii) myeloid cells/µl peripheral blood of C57BL/6 mice bearing Panc02 tumor left untreated (NT) or treated beginning on day 14 with 3 daily doses of 20 Gy focal radiation (RT). In each graph, each symbol represents one mouse. NS = Not significant; * = p<0.05; ** = p<0.01; *** = p<0.005; **** = p<0.001. Data represents multiple replicate experiments.

The expansion of myeloid populations is associated with cancer progression in animal models and in cancer patients. In mice, the myeloid expansion varies between tumor models, for example with particularly pronounced myeloid expansions described in the 4T1 mammary carcinoma model [18]. In these models, mice bearing advanced tumors exhibit extremely large spleens, containing a particularly noticeable expansion in the CD11b^+^Gr1^+^ population. To track myeloid expansion in mice through tumor growth without the need to euthanize the animal we monitored myeloid populations in the peripheral blood. Standard preparation of peripheral blood mononuclear cells (PBMC) by density gradient centrifugation can lead to loss of granulocyte populations, which make up the majority of the myeloid cells in peripheral blood. Therefore, we developed a whole blood flow cytometry assay incorporating count-beads to measure absolute numbers of circulating myeloid cells. Repeated measures of multiple mice demonstrated that this myeloid population expanded by 2–3 logs through tumor growth, dramatically increasing the overall cellularity of peripheral blood. In naïve mice the CD11b^+^ myeloid cells broadly consisted of Gr1^+^ and Gr1^−^ cells (**Figure 1a**), where the CD11b^+^Gr1^+^ cells were uniformly MHC class II (IA) negative and the CD11b^+^Gr1^−^ cells incorporated both MHC class II negative and positive cells (**Figure 1aii**). In 4T1 tumor-bearing mice we observed similar phenotypes, but a large increase in the number of these cells, notably an expansion in CD11b^+^Gr1^+^ cells, consistent with the observations of other investigators was observed [4], [19]. Radiation therapy of mice bearing established 4T1 tumors controls tumor growth; however despite high radiation doses, the tumors recur locally (**Figure 1b**). Radiation therapy of the primary tumor resulted in a significant difference in peripheral myeloid cells, compared to untreated mice, within one week of radiation therapy (p<0.01 day 21 following tumor challenge) (**Figure 1b**). At this point, the numbers of circulating myeloid cells in treated mice were significantly lower than pre-treatment levels for a little over one week before expansion returned (RT d14 vs. RT d21 (p<0.05), RT d14 vs. RT d23 (p<0.05), RT d14 vs. RT d27 (p = 0.26)). The numbers of circulating myeloid cells in treated mice remained significantly lower than in untreated mice until approximately two weeks post-treatment (p<0.05 day 27 following tumor challenge). At this point, recurrence of the primary tumor was evident (**Figure 1bi**) suggesting a potential link between primary tumor burden and myeloid numbers. These data demonstrate that radiation therapy of the primary tumor transiently reverses the tumor-associated myeloid expansion.

The 4T1 model is spontaneously metastatic, predominantly to the lungs. Metastases are seeded within 10 days of primary tumor implantation – after this time surgical removal of the primary tumor does not cure mice, but instead eventually results in mortality due to progressive growth of metastatic disease [20]. To monitor metastatic progression in our radiation therapy model, we harvested lungs from mice that required euthanasia due to their primary tumor exceeding 12 mm, or due to poor condition. In mice treated with radiation therapy 14 days after tumor challenge, control of the primary tumor resulted in delayed lung harvest compared to untreated mice. However, on lung harvest, clonogenic analysis demonstrated that the metastases continued to progress (**Figure 1ci**). Even in mice where the radiation-treated tumor was stable, or progressing only slowly, within 35–50 days of challenge all mice became sufficiently unwell to require euthanasia, and this morbidity was universally associated with an extensive burden of lung disease. These data confirm extensive historical data that focal radiation therapy of the primary tumor is a highly local therapy resulting in cancer control exclusively in the radiation field: in the hundreds of thousands of patients treated per year with radiation therapy, abscopal effects – treatment induced control of tumors outside the radiation field – are extremely rare where radiation is the only treatment modality [21]. Since myeloid expansion in patients has been associated with invasive and metastatic disease [5], it is possible that the residual metastatic disease prevents full normalization of myeloid numbers following local therapy. To examine the contribution of metastatic disease to myeloid expansion, mice were injected with 4T1 tumors intravenously to directly form metastases in the absence of a primary tumor. Mice bearing only metastatic disease exhibited peripheral myeloid expansion (**Figure 1cii**), but there were substantially fewer myeloid cells present than in mice with synchronous sub-cutaneous primary tumors. This relationship was sustained in the spleen, where mice bearing only metastases exhibited significantly more cells than non-tumor bearing mice, but this was significantly fewer than mice with sub-cutaneous primary tumors (**Figure 1ciii**). Since we know that focal radiation therapy controls the primary tumor locally but does not control metastases, mice receiving radiation therapy retain both their residual local disease *and* their lung tumor burden. Therefore these data are consistent with the combination of residual local and metastatic disease preventing myeloid numbers returning to baseline following radiation therapy, and is consistent with observations following chemotherapy [9] and surgical excision [9], [12].

This effect of radiation therapy was not limited to the 4T1 model. The Panc02 pancreatic adenocarcinoma tumor model drives myeloid expansion with tumor progression, though to a lesser extent than 4T1 (**Figure 1di**). Radiation therapy of Panc02 tumors caused a transient control of tumor growth (**Figure 1dii**), followed by an aggressive outgrowth [22]. Like treatment of 4T1, radiation therapy to Panc02 tumors resulted in a significant decrease in peripheral myeloid cells (**Fig. 1diii**). Together, these data demonstrate that cytotoxic therapy targeted at the primary tumor causes a systemic, though transient reversal of the myeloid expansion driven by tumor growth.

**Figure 2:**
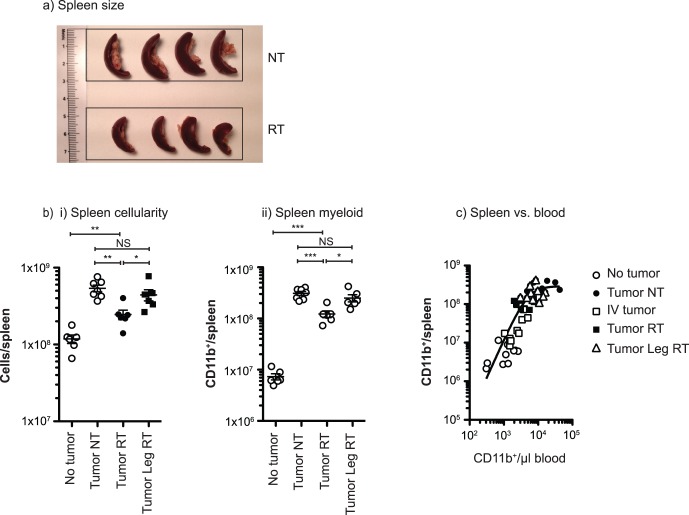
The splenic response to tumor radiation. a) Freshly excised spleens from d24 4T1 tumor-bearing BALB/c mice left untreated (NT) or treated beginning on day 14 with 3 daily doses of 20 Gy focal radiation (RT). Boxes shown are 3 cm wide. b) i) Total spleen cellularity in naïve mice (No tumor) and mice day 24 following injection of 4T1 left untreated (Tumor NT) or treated beginning on day 14 with 3 daily doses of 20 Gy focal radiation to the tumor (Tumor RT) or to the uninvolved opposite limb (Tumor Leg RT). ii) The number of CD11b^+^ cells per spleen in mice from the experiment shown in i). c) The number of CD11b^+^ cells in the spleen plotted against the number of CD11b^+^ cells/µl peripheral blood at harvest for each tumor and treatment group.

At the nadir of blood myeloid cells following radiation therapy, the spleens were visibly smaller (**Figure 2a**). We counted cells in the spleen as a measure of myeloid contraction following radiation therapy, and while total spleen cellularity declined seven days following radiation therapy, it remained at an intermediate size – significantly less cells than mice with no treatment (p<0.01) and significantly more cells than mice without tumors (p<0.01) (**Figure 2bi**). To determine whether the decrease in systemic myeloid numbers was caused by radiation treatment independently of effects on the tumor, mice bearing 4T1 tumors were treated with radiation to the contralateral non tumor-bearing leg. Mice receiving radiation therapy to the tumor displayed significantly fewer total cells in the spleen than untreated mice or those treated on the contralateral limb (**Figure 2bi**). CD11b^+^ cells were by far the largest population in the expanded spleen of tumor-bearing mice, and the CD11b^+^ population in the spleen showed a similar intermediate result following radiation therapy of the primary tumor, with mice treated with tumor radiation therapy exhibiting significantly fewer CD11b^+^ cells than untreated mice or mice irradiated on the non-tumor-bearing leg (**Figure 2bii**). Again, despite the reduction caused by tumor radiation, treated mice retained a significant elevation in myeloid cells over non-tumor bearing mice and there was no significant difference between untreated mice and mice irradiated to the non-treatment leg (**Figure 2bii**). The similar myeloid response to radiation of the tumor seen in the peripheral blood and in the spleen results in a close correlation between these measures regardless of tumor or treatment status (**Figure 2c**). Since radiation therapy to the tumor and to the opposite limb will irradiate the blood pool through treatment, that radiation therapy delivered to the opposing limb is does not reduce myeloid numbers in the spleen indicates that the effect on myeloid populations is not due to direct effects of radiation on myeloid cells or any potential scatter-doses.

**Figure 3:**
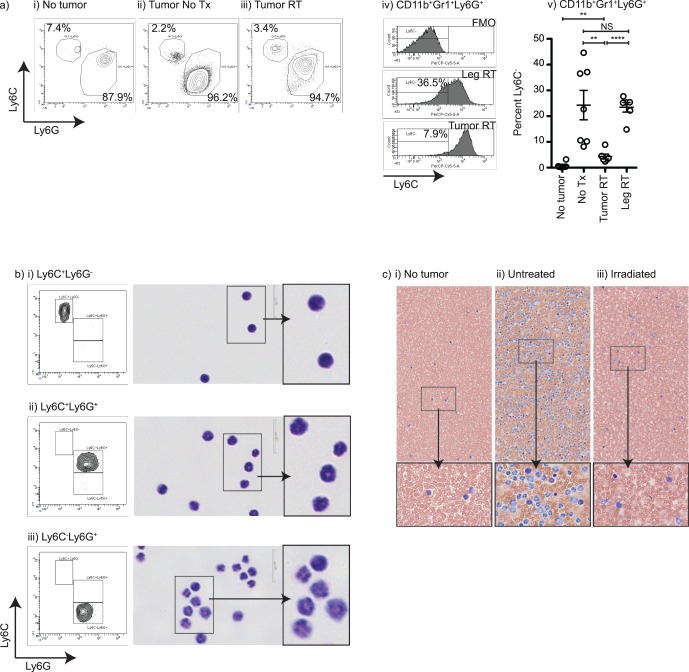
Myeloid subpopulations responding to tumor radiation. a) Flow cytometry of fresh whole peripheral blood from i) naïve mice or mice bearing 4T1 tumors ii) left untreated or iii) irradiated, showing Ly6C and Ly6G within gated CD11b^+^Gr1^hi^ myeloid populations. iv) Histograms of Ly6C expression in gated CD11b^+^Gr1^hi^Ly6G^+^ cells, including negative control staining (FMO) and showing the percentage Ly6C^−^ in mice irradiated in the tumor (Tumor RT) or the opposite limb (Tumor Leg RT). v) The percentage of CD11b^+^Gr1^hi^Ly6G^+^ cells that are Ly6C^−^ from naïve mice (No tumor) and mice day 24 following injection of 4T1 left untreated (Tumor NT) or treated beginning on day 14 with 3 daily doses of 20Gy focal radiation to the tumor (Tumor RT) or to the uninvolved opposite limb (Tumor Leg RT). Each symbol represents one mouse. b) Cytospins of sorted CD11b^+^Gr1^hi^ cells from untreated mice that are i) Ly6C^+^Ly6G^−^, ii) Ly6C^+^Ly6G^+^, or iii) Ly6C^−^Ly6G^+^. Each cytospin is shown next to the sort purity plot with increased magnification on the inset box. c) Wright-Giemsa stain of d24 blood smears from i) naïve mice (No tumor) and mice day 24 following injection of 4T1 ii) left untreated (Tumor NT) or iii) treated beginning on day 14 with 3 daily doses of 20 Gy focal radiation to the tumor (Tumor RT). The inset box is rotated and shown at increased magnification. NS = Not significant; * = p<0.05; ** = p<0.01; *** = p<0.005; **** = p<0.001. Data represents multiple replicated experiments; each subfigure includes data from a different replicate experiment.

To determine whether any specific myeloid population was particularly affected by radiation therapy, we performed additional sub-phenotyping of peripheral blood myeloid cells. The Gr1 antibody recognizes both Ly6G and Ly6C, and antibodies to these markers were used to distinguish subpopulations within CD11b^+^ cells [6]. Consistent with the literature, within the CD11b^+^Gr1^+^ cells were two major populations distinguished by Ly6C and Ly6G: a clearly distinct population of Ly6G^−^Ly6C^+^ cells and a large population of Ly6G^+^ cells that displayed varying expression of Ly6C (**Figure 3ai–iii**). Following radiation therapy, there was an apparent loss of CD11b^+^Gr1^+^Ly6G^+^ cells expressing lower levels of Ly6C (**Figure 3aiii)**. Using controls to identify Ly6G^+^Ly6C^−^ cells (**Figure 3aiv**) we demonstrated that radiation therapy to the tumor resulted in a significant decrease in CD11b^+^Gr1^+^Ly6G^+^ cells that were Ly6C^−^. This did not occur when the opposite limb was irradiated (**Figure 3av**). To characterize these populations, we sorted these sub-phenotypes from the peripheral blood of mice bearing 4T1 tumors (**Figure 3b**). In agreement with previous characterizations [6], Ly6G^−^Ly6C^+^ cells had monocyte morphology, Ly6G^+^Ly6C^+^ had the morphology of neutrophils and Ly6G^+^Ly6C^−^ cells had the morphology of mature neutrophils. Similarly, blood smears from tumor-bearing mice demonstrated a marked expansion in neutrophils, which were greatly diminished following radiation therapy to the tumor (**Figure 3c**). These data demonstrate that radiation therapy of the tumor results in a particular reversal of tumor-driven neutrophil expansion.

**Figure 4:**
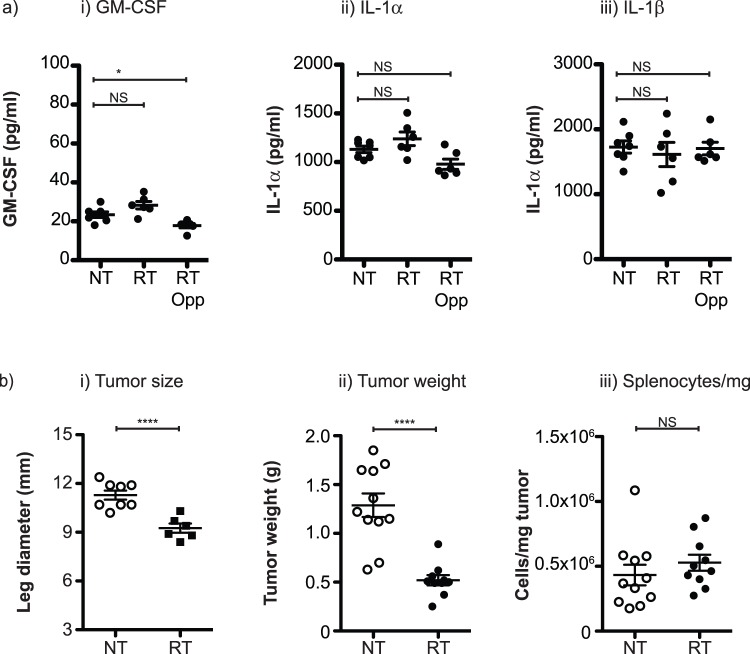
Tumor cytokine and growth factor levels following radiation therapy. a) Bead assay for i) GM-CSF, ii) IL-1α and iii) IL-1β in homogenates of 4T1 tumors at day 24 following injection of 4T1 left untreated (NT) or treated beginning on day 14 with 3 daily doses of 20 Gy focal radiation to the tumor (RT) or to the uninvolved opposite limb (RT Opp). b) d24 following tumor challenge graphs show i) leg diameter ii) tumor weight and iii) splenocytes per spleen/tumor weight for mice left untreated (NT) or treated beginning on day 14 with 3 daily doses of 20 Gy focal radiation (RT). In each graph, each symbol represents one mouse. NS = Not significant; * = p<0.05; ** = p<0.01; *** = p<0.005; **** = p<0.001. Data is representative of three replicate experiments.

Tumor-driven myeloid expansions are linked to the local inflammatory environment and engineered expression of GM-CSF [23] or IL-1β [24] by cancer cells has been shown to drive myeloid expansion. To determine whether radiation therapy influenced myeloid numbers by modulating these cytokines, we analyzed their levels in the tumor. The levels of GM-CSF, IL-1α, and IL-1β were not altered in the tumor following radiation therapy (**Figure 4a**). These data indicate that the balance of these inflammatory cytokines and growth factors in the tumor is not directing the change in myeloid numbers. While we cannot rule out regulation of other growth factors, it is perhaps more relevant that following radiation therapy the primary tumors are significantly smaller by diameter (**Figure 4bi**) and weight (**Figure 4bii**). If we calculate the number of cells in the spleen per mg of primary tumor, there is no difference between untreated and irradiated mice (**Figure 4biii**). Thus, even without growth factor regulation at the tumor site, a smaller tumor burden will mean fewer tumor-derived growth factors to influence myeloid numbers. Therefore, these data suggest that myeloid contraction following cytotoxic and cytoreductive therapy is determined primarily by fluctuations in the number of cancer cells.

**Figure 5:**
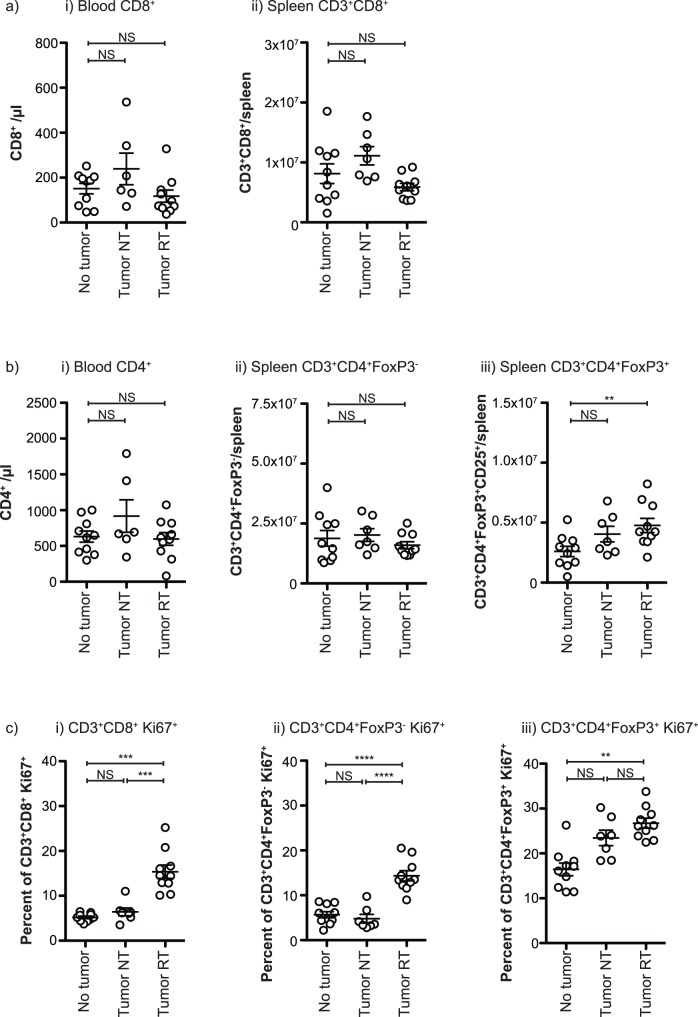
T cell activity in spleens during myeloid contraction. a) The number of i) CD8/µl peripheral blood or ii) the number of CD3^+^CD8^+^ cells in the spleen of: naïve mice (No tumor); mice bearing d24 4T1 tumor-bearing BALB/c mice left untreated (Tumor NT); or mice treated beginning on day 14 with 3 daily doses of 20 Gy focal radiation to the tumor (Tumor RT). b) The number of i) CD4/µl peripheral blood; ii) the number of non-regulatory CD3^+^CD4^+^FoxP3^−^ T cells in the spleen; and iii) the number of CD3^+^CD4^+^FoxP3^+^ T regulatory cells in the spleen of mice grouped as in a). c) Expression of Ki67 in i) gated CD3^+^CD8^+^ cells ii) gated non-regulatory CD3^+^CD4^+^FoxP3^−^ T cells or iii) gated CD3^+^CD4^+^FoxP3^+^ T regulatory cells in the spleen of mice grouped as in a). In each graph, each symbol represents one mouse. NS = Not significant; * = p<0.05; ** = p<0.01; *** = p<0.005; **** = p<0.001. Data represents combined data from 2 replicate experiments. Representative plots for these data are found in **Figure S1**.

Subpopulations of myeloid cells from tumor-bearing mice can exert suppressive effects on T cell function *in vitro*. For this reason we examined T cells in the spleen following radiation therapy. Due to myeloid expansion in the spleen, T cells make up a smaller percentage of splenocytes; however total CD8 T cell number was not different in tumor bearing mice or following radiation therapy of the tumor (**Figure 5a**). That CD8 T cells remain constant while myeloid cells increase results in a dramatically skewed myeloid: T cell ratio – in the spleen the ratio increases from a mean of approximately 1.7 CD11b^+^ per CD8^+^ to 29 CD11b^+^ per CD8^+^ (p<0.001). The result of the radiation-induced decline in myeloid cells is an improvement in the myeloid: CD8 ratio in the spleen – to 20 CD11b^+^ per CD8^+^ (p<0.01) – suggesting a somewhat improved potential to initiate *de novo* immune responses. Consistent with the data in CD8 T cells, there was no change in the number of non-regulatory CD4 T cells in tumor-bearing mice and treated mice compared to controls (**Figure 5b**). However, tumor burden and radiation therapy is accompanied by an increase in the number of T regulatory cells (T^reg^) in the spleen (**Figure 5b**), as measured by CD4^+^ cells expressing CD25 and FoxP3 (**Figure S1**). Interestingly, radiation therapy significantly increased proliferation of CD8 T cells and non-regulatory CD4 T cells (**Figure 5c**) as measured by expression of Ki67 (**Figure S1**), suggesting that endogenous immune responses may be more active in the spleen following radiation therapy of the primary tumor. These data suggest that there is increased adaptive immune activity in the spleen following radiation therapy that correlates with myeloid contraction.

**Figure 6:**
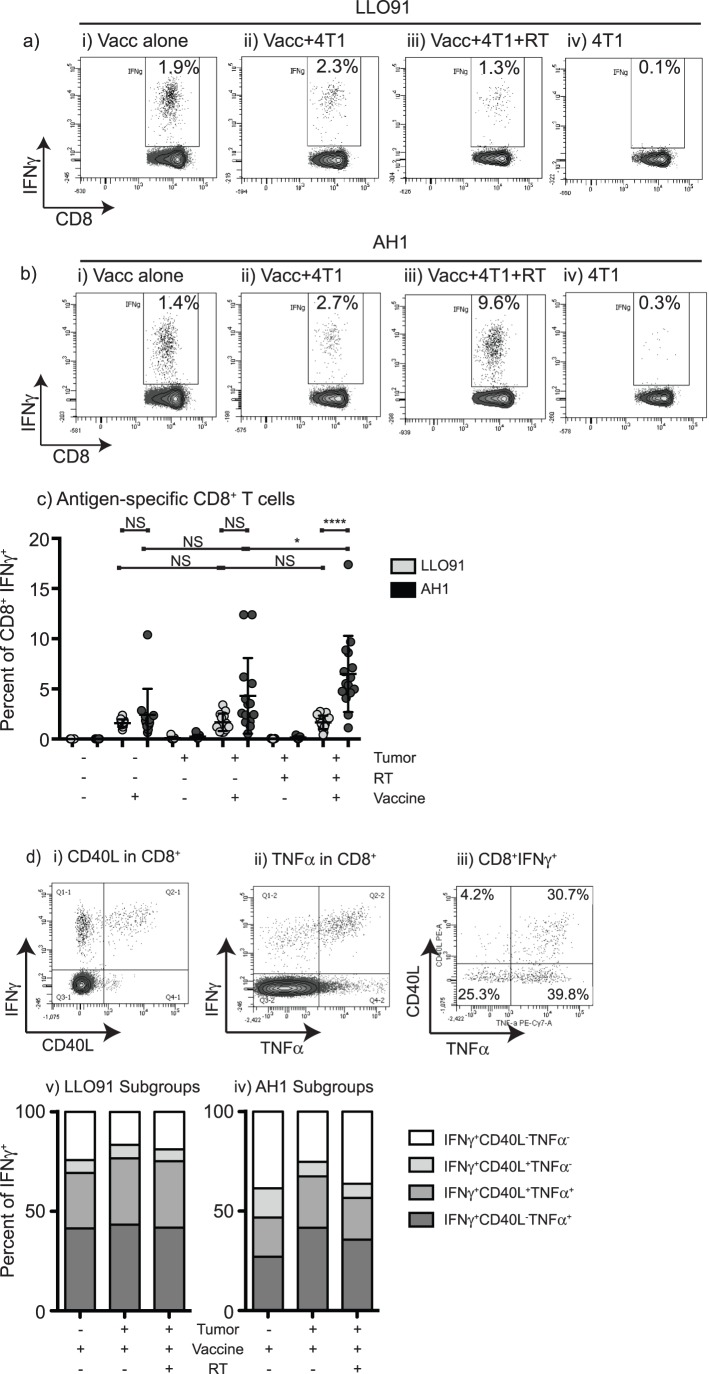
Listeria vaccination of mice during myeloid contraction. Flow cytometry of intracellular IFNγ production in response to a) LLO91 or b) AH1 peptide, from mouse spleens 7 days following Listeria vaccination of i) naïve mice, ii) tumor bearing mice left untreated, iii) tumor bearing mice with their tumor irradiated prior to vaccination, iv) control tumor-bearing mice not receiving vaccination. c) Summary of intracellular IFNγ production in response to each peptide, each symbol represents one mouse. Data represents combined data from 3 replicate experiments. d) representative staining showing i) dual IFNγ-CD40L, ii) dual IFNγ-TNFα or iii) triple IFNγ-CD40L-TNFα positive cells from vaccinated mice in response to LLO91 peptide. Summary of single, dual and triple positive IFNγ^+^ cells from each vaccinated group stimulated with iv) LLO91 and v) AH1 peptides. NS = Not significant; * = p<0.05; ** = p<0.01; *** = p<0.005; **** = p<0.001.

While radiation therapy may improve the splenic environment by decreasing myeloid cells, these assays cannot distinguish whether T cell proliferation occurs as a result of decreased myeloid cells or due to other factors – for example, increased release of antigens following radiation therapy. To formally test whether the changed splenic environment following radiation therapy influenced the ability to mount a new immune response, we tested the ability of mice to respond to vaccination. Intravenously administered *L. monocytogenes* primarily infects macrophages in the spleen and Kuppfer cells in the liver, and results in a robust antigen-specific cellular immune response. The splenic focus and myeloid-targeting makes the *L. monocytogenes* vaccine platform ideal to monitor the consequence of myeloid expansion on T cell responses in the spleen. We used a live-attenuated *L. monocytogenes*-based vaccine expressing an altered peptide ligand of the class I -restricted peptide AH1, which is derived from the endogenous self-antigen gp70 that is also expressed by 4T1 cancer cells [25]. As a control, we measured responses to the *L. monocytogenes*-derived MHC class I binding LL0_91–99_ peptide which is not tumor-associated and to which the mice are naïve [**26**]. Mice were immunized one day following the final dose of radiation (day 17 following tumor challenge). Untreated mice and non-tumor-bearing mice were immunized on the same day. Spleens were harvested seven days following vaccination and the frequency of LLO_91–99_- (**Figure 6a**) and AH1-specific (**Figure 6b**) CD8+ T cells was determined by intracellular cytokine staining. Interestingly, the CD8+ T cell response to the LL0 was the same in tumor-free mice and in mice bearing 4T1 tumors, despite the pronounced myeloid expansion in the spleen of tumor-bearing mice (**Figure 6c**). Mice receiving tumor radiation, which resulted in decreased numbers of myeloid cells, did not exhibit any changes in the LLO-specific CD8+ T cell response relative to tumor-free mice or to mice with untreated tumors. These data indicate that the antigen-specific CD8+T cell response is not globally suppressed by the tumor-associated expansion of myeloid cells. In the absence of vaccination, radiation therapy of tumor-bearing mice did not significantly alter the T cell response to AH1 (**Figure 6c**). However, in mice receiving vaccination the AH1 response was significantly increased by tumor radiation, resulting in a significant increase relative to vaccine alone or radiation alone (**Figure 6c**). To assess the quality of the response, we evaluated CD40L and TNFα production within the IFNγ^+^ antigen-specific CD8^+^ T cell population. In the LLO91 response, a proportion of the IFNγ positive cells also expressed CD40L or TNFα (**Figure 6c**). The proportions of double and triple-positive LLO91-specific T cells was not influenced by the presence of tumor-induced myeloid expansion, and was not altered by radiation therapy (**Figure 6d**). Thus despite the published suppressive effect of splenic myeloid cells *ex vivo*, these data demonstrate that the splenic myeloid expansion does not influence the degree or quality of the *in vivo* T cell response to *L. monocytogenes*-associated neoantigens. In addition, these data demonstrate that radiation therapy to the primary tumor significantly increases the vaccine directed response to antigens associated with the tumor.

## Discussion

These data demonstrate that radiation therapy of the tumor halts the myeloid expansion associated with tumor growth, but this myeloid expansion is restored following recurrence of the primary tumor (**Figure 1**). Myeloid numbers do not return to baseline following radiation therapy, but remain elevated due to the contribution of residual local and metastatic disease, and myeloid contraction in the blood is matched in the spleen (**Figure 2**). While total myeloid numbers decline, radiation therapy of the tumor causes a particular decline in the expanded population of mature neutrophils (**Figure 3**). Myeloid contraction is not caused by radiation-mediated regulation of GM-CSF and IL-1, which have been shown to drive myeloid expansion [18], [23], [27]; rather, myeloid numbers closely follow tumor size (**Figure 4**). Although T cell numbers remain constant in the spleen and peripheral blood during myeloid expansion, following radiation therapy and myeloid contraction there is an increase in CD8 and non-regulatory CD4 T cell proliferation (**Figure 5**), suggesting an improved T cell activation environment. The myeloid expansion associated with tumor growth does not suppress the *in vivo* T cell response to novel antigens presented via Listeria vaccination; therefore, the myeloid contraction caused by radiation therapy of the tumor does not improve the response to vaccination (**Figure 6**). However, T cell antigen-specific responses to self-antigens expressed in the vaccine and in the irradiated tumor are increased following radiation therapy. These data demonstrate for the first time that radiation therapy can reverse the myeloid expansion associated with tumor growth. These data closely correlate with prior data in surgical and chemotherapy models, and it is interesting to propose that where myeloid expansion is measurable, myeloid normalization could be evaluated as a read-out for *in vivo* cytoreductive efficacy.

Invasive tumors exhibit significantly more myeloid expansion than tumors of lower stage and pre-malignant tumors in patients [5] and in murine transgenic models of cancer progression [28], [29]. In transplantable tumor models, where the cancer cells reproducibly generate particular growth and invasion patterns, the association of myeloid expansion is model-specific. For example, the spontaneously metastatic BALB/c 4T1 mammary carcinoma is associated with dramatic myeloid expansion, while the spontaneously metastatic C57BL/6 B16D5 model causes a minimal myeloid expansion. It is notable that when comparing the 4T1 and CL66 variants of the same BALB/c mammary carcinoma [14], that each is spontaneously metastatic but CL66 does not result in a myeloid expansion [30]. Thus, myeloid expansion may be associated with invasive disease, and myeloid cells may participate in the metastatic process, but it is not clear that myeloid *expansion* is required for tumor invasion and metastases. Peripheral blood monocytes and granulocytes are dependent to varying degrees on the presence of the growth factors M-CSF, G-CSF and GM-CSF for *in vivo* expansion and differentiation from the bone marrow [8]. The myeloid expansion associated with cancer has been linked to inflammation in the tumor [18]; however, inflammatory cytokines do not have the capacity to act as growth factors capable of driving myeloid expansion from bone marrow precursors. Instead, inflammatory cytokines including IL-1 and TNFα are strong inducers of growth factor production by stromal cells [31], endothelial cells [32], and cancer cells [33] as well as monocytes [34] and T cells [35]. Thus, while proinflammatory cytokine production by cancer cells is associated with myeloid expansion, the mechanism of myeloid expansion is of necessity via growth factor induction. While we do not see changes in GM-CSF expression in the tumor, G-CSF expression is particularly associated with models causing extreme myeloid expansions [19], [30], and in these models blockade of G-CSF and not GM-CSF or M-CSF has been shown to decrease accumulation of Ly6G^+^ cells in tumors and lung metastases [19]. This contribution of G-CSF may explain the high proportion of neutrophils in cancer-driven myeloid expansion [36]. In the 4T1 model G-CSF and not GM-CSF was detectable in the blood of tumor-bearing mice, and levels correlated with tumor progression [37]. Our data demonstrates that while the level of GM-CSF, IL-1α and IL-1β per mg of tumor does not change following radiation therapy, the decrease in size of the tumor will result in fewer of these and other tumor-derived growth factors in the tumor-bearing mouse following radiation therapy. In this way, tumor debulking through radiation or other therapies causes a decrease in tumor-derived growth factor and cytokine levels in the treated animal.

Despite the dysfunction in the tumor environment and in systemic immune populations, when tested with standard vaccines there is little evidence that cancer patients are functionally immunosuppressed [38]. Our data with Listeria vaccination agrees with these clinical studies. Depletion of myeloid cells or redirected myeloid differentiation may be most relevant at the tumor, since the most consistent biological effect of targeting myeloid cells is increased tumor control associated with increased T cell numbers and effector function in the tumor [39], [40], [41]. In our model the proliferation of T cells in the spleen following radiation therapy likely depends on T cell stimulation by antigen, which may be released by treatment, but it is also likely that the eventual effector function of those T cells is enhanced because there are fewer myeloid cells. However, since the response to Listeria-restricted antigens is not influenced by the tumor or enhanced by radiation therapy, either the vaccine response is unaffected by the myeloid expansion and contraction or the Listeria vaccine platform is resistant to tumor-associated myeloid cells. Published studies using *in vivo* vaccination with cell-based and DNA vaccines [42], or vaccinia-based vectors [43] incorporating model tumor antigens have shown that antigen-specific responses are inhibited by tumor-associated myeloid expansions. The difference may be that Ly6C^+^ Ly6G^−^ monocytic cells are critical for immune responses to Listeria, and Ly6G^+^ neutrophils do not play a positive or a negative role [44]. These Ly6C^+^ Ly6G^−^ monocytic cells are also more suppressive than Ly6G^+^ MDSC in assays of *in vitro* T cell proliferation [6], [27]. Thus, Listeria-based vaccines may be a superior approach in cancer patients since Listeria infection specifically targets these potentially suppressive monocytic cells and is not affected by neutrophil expansions. The relative contribution of antigen and reduced myeloid cells remains to be determined, but in either case there is likely a finite window following radiation therapy to take advantage of these factors. Recent data demonstrates that the combination of listeria vaccination and tumor radiation in a murine prostate cancer model resulted in increased numbers of tumor antigen-specific T cells and increased tumor control compared to either treatment alone [45]. Our very similar T cell data with endogenous antigen suggests that Listeria-based vaccines may be particularly good partners for radiation therapy. In addition to antigen release, radiation therapy may cause release of endogenous adjuvants and antigens both from normal tissue and cancer-associated tissues. This could result in antigen-specific responses against new tumor-associated antigens but also could engender autoimmune responses. The combination of radiation therapy with a potent vaccine may be an effective technique to focus immune activity on immunodominant targets in the tumor while taking advantage of the ability of radiation therapy to improve the tumor site as a target for effector activity [46], [47]. Vaccination has shown significant efficacy in combination with radiation therapy [46], [48] and a strong vaccine may be an important tool to overcome tolerance to the tumor-associated antigens that are released by treatment. Thus, while radiation therapy does not currently result in frequent abscopal effects when delivered alone, there is great potential for abscopal cures when radiation therapy is combined with immunotherapy [16].

It is interesting to note that those murine tumors in which myeloid expansions are most notable and most studied: namely 4T1; EMT6; 3LL; CT26; and EL4, include spontaneously metastatic and non-metastatic primary tumors, encompass both immunogenic and poorly immunogenic tumors, but all are either dependent on functional adaptive immunity for the full effect of radiation therapy [49], or have been more effectively treated by a combination of radiation with immunotherapy than by immunotherapy or radiation alone [50], [51], [52], [53], [54]. We propose that radiation therapy of the tumor, through some combination of cytoreduction, release of antigen and adjuvant, and changes in the local immune environment, provides a window of opportunity for immunotherapy. Listeria vaccines, which are not affected by myeloid expansion and effectively prime high quality T cell responses in tumor bearing mice, have potential to direct immune responses to target residual disease following radiation therapy of tumors, and we are further studying their combination with radiation therapy.

## Supporting Information

Figure S1 **Representative Flow Plots during myeloid contraction.** a) Staining for FoxP3^+^CD25^+^ T regulatory cells in gated CD3^+^CD4^+^ cells in the spleen showing staining i) the absence of CD25 antibody, ii) the absence of FoxP3 antibody. Examples of FoxP3^+^CD25^+^ T regulatory cells are shown for iii) naïve mice or 4T1 tumor-bearing mice receiving radiation to the tumor. b) Expression of Ki67 in gated CD3^+^CD8^+^ cells showing i) the absence of Ki67 antibody and examples of Ki67 expression by CD3^+^CD8^+^ T cells in the spleen of ii) naïve mice (No tumor) or iii) mice bearing d24 4T1 tumor-bearing BALB/c mice treated beginning on day 14 with 3 daily doses of 20 Gy focal radiation to the tumor (Tumor RT). c) Expression of Ki67 in gated CD3^+^CD4^+^ cells as per b), showing FoxP3 on the x-axis to distinguish non-regulatory and regulatory T cells. Numbers represent the percentage of CD3^+^CD4^+^FoxP3^−^ or CD3^+^CD4^+^FoxP3^+^ cells that are Ki67^+^ rather than the percentage of all CD3^+^CD4^+^ cells.
(EPS)

## References

[1] Lin EY, Nguyen AV, Russell RG, Pollard JW (2001) Colony-stimulating factor 1 promotes progression of mammary tumors to malignancy. J Exp Med 193: 727–740. 11257139 10.1084/jem.193.6.727 PMC2193412

[2] Lin EY, Li JF, Gnatovskiy L, Deng Y, Zhu L, et al (2006) Macrophages regulate the angiogenic switch in a mouse model of breast cancer. Cancer Res 66: 11238–11246. 17114237 10.1158/0008-5472.CAN-06-1278

[3] DeNardo DG, Brennan DJ, Rexhepaj E, Ruffell B, Shiao SL, et al (2011) Leukocyte Complexity Predicts Breast Cancer Survival and Functionally Regulates Response to Chemotherapy. Cancer Discovery 1: 54–67. 22039576 10.1158/2159-8274.CD-10-0028 PMC3203524

[4] Gabrilovich DI, Bronte V, Chen SH, Colombo MP, Ochoa A, et al.. (2007) The terminology issue for myeloid-derived suppressor cells. Cancer Res 67: 425; author reply 426. 10.1158/0008-5472.CAN-06-3037 PMC1941787 17210725

[5] Diaz-Montero CM, Salem ML, Nishimura MI, Garrett-Mayer E, Cole DJ, et al (2009) Increased circulating myeloid-derived suppressor cells correlate with clinical cancer stage, metastatic tumor burden, and doxorubicin-cyclophosphamide chemotherapy. Cancer Immunol Immunother 58: 49–59. 18446337 10.1007/s00262-008-0523-4 PMC3401888

[6] Movahedi K, Guilliams M, Van den Bossche J, Van den Bergh R, Gysemans C, et al (2008) Identification of discrete tumor-induced myeloid-derived suppressor cell subpopulations with distinct T cell-suppressive activity. Blood 111: 4233–4244. 18272812 10.1182/blood-2007-07-099226

[7] Bayne LJ, Beatty GL, Jhala N, Clark CE, Rhim AD, et al (2012) Tumor-derived granulocyte-macrophage colony-stimulating factor regulates myeloid inflammation and T cell immunity in pancreatic cancer. Cancer Cell 21: 822–835. 22698406 10.1016/j.ccr.2012.04.025 PMC3575028

[8] Gabrilovich DI, Nagaraj S (2009) Myeloid-derived suppressor cells as regulators of the immune system. Nature reviews Immunology 9: 162–174. 10.1038/nri2506 PMC2828349 19197294

[9] Sinha P, Clements VK, Bunt SK, Albelda SM, Ostrand-Rosenberg S (2007) Cross-talk between myeloid-derived suppressor cells and macrophages subverts tumor immunity toward a type 2 response. J Immunol 179: 977–983. 17617589 10.4049/jimmunol.179.2.977

[10] Vincent J, Mignot G, Chalmin F, Ladoire S, Bruchard M, et al (2010) 5-Fluorouracil selectively kills tumor-associated myeloid-derived suppressor cells resulting in enhanced T cell-dependent antitumor immunity. Cancer Research 70: 3052–3061. 20388795 10.1158/0008-5472.CAN-09-3690

[11] Suzuki E, Kapoor V, Jassar AS, Kaiser LR, Albelda SM (2005) Gemcitabine selectively eliminates splenic Gr-1+/CD11b+ myeloid suppressor cells in tumor-bearing animals and enhances antitumor immune activity. Clinical cancer research : an official journal of the American Association for Cancer Research 11: 6713–6721. 16166452 10.1158/1078-0432.CCR-05-0883

[12] Sinha P, Clements VK, Ostrand-Rosenberg S (2005) Reduction of myeloid-derived suppressor cells and induction of M1 macrophages facilitate the rejection of established metastatic disease. J Immunol 174: 636–645. 15634881 10.4049/jimmunol.174.2.636

[13] Makarenkova VP, Bansal V, Matta BM, Perez LA, Ochoa JB (2006) CD11b+/Gr-1+ myeloid suppressor cells cause T cell dysfunction after traumatic stress. J Immunol 176: 2085–2094. 16455964 10.4049/jimmunol.176.4.2085

[14] Aslakson CJ, Miller FR (1992) Selective events in the metastatic process defined by analysis of the sequential dissemination of subpopulations of a mouse mammary tumor. Cancer Research 52: 1399–1405. 1540948

[15] Priebe TS, Atkinson EN, Pan BF, Nelson JA (1992) Intrinsic resistance to anticancer agents in the murine pancreatic adenocarcinoma PANC02. Cancer Chemother Pharmacol 29: 485–489. 1348974 10.1007/BF00684853

[16] Seung SK, Curti BD, Crittenden M, Walker E, Coffey T, et al (2012) Phase 1 Study of Stereotactic Body Radiotherapy and Interleukin-2‚ÄîTumor and Immunological Responses. Science Translational Medicine 4: 137ra174. 10.1126/scitranslmed.3003649 22674552

[17] Brockstedt DG, Giedlin MA, Leong ML, Bahjat KS, Gao Y, et al (2004) Listeria-based cancer vaccines that segregate immunogenicity from toxicity. Proceedings of the National Academy of Sciences of the United States of America 101: 13832–13837. 15365184 10.1073/pnas.0406035101 PMC518841

[18] Bunt SK, Yang L, Sinha P, Clements VK, Leips J, et al (2007) Reduced inflammation in the tumor microenvironment delays the accumulation of myeloid-derived suppressor cells and limits tumor progression. Cancer Res 67: 10019–10026. 17942936 10.1158/0008-5472.CAN-07-2354 PMC4402704

[19] Kowanetz M, Wu X, Lee J, Tan M, Hagenbeek T, et al (2010) Granulocyte-colony stimulating factor promotes lung metastasis through mobilization of Ly6G+Ly6C+ granulocytes. Proceedings of the National Academy of Sciences of the United States of America 107: 21248–21255. 21081700 10.1073/pnas.1015855107 PMC3003076

[20] Tamai H, Watanabe S, Zheng R, Deguchi K, Cohen PA, et al (2008) Effective treatment of spontaneous metastases derived from a poorly immunogenic murine mammary carcinoma by combined dendritic-tumor hybrid vaccination and adoptive transfer of sensitized T cells. Clinical Immunology 127: 66–77. 18262845 10.1016/j.clim.2007.12.001

[21] Kaminski JM, Shinohara E, Summers JB, Niermann KJ, Morimoto A, et al (2005) The controversial abscopal effect. Cancer treatment reviews 31: 159–172. 15923088 10.1016/j.ctrv.2005.03.004

[22] Crittenden MR, Cottam B, Savage T, Nguyen C, Newell P, et al (2012) Expression of NF-kappaB p50 in Tumor Stroma Limits the Control of Tumors by Radiation Therapy. PLoS ONE 7: e39295. 22761754 10.1371/journal.pone.0039295 PMC3386283

[23] Bronte V, Chappell DB, Apolloni E, Cabrelle A, Wang M, et al (1999) Unopposed production of granulocyte-macrophage colony-stimulating factor by tumors inhibits CD8+ T cell responses by dysregulating antigen-presenting cell maturation. J Immunol 162: 5728–5737. 10229805 PMC2228333

[24] Bunt SK, Sinha P, Clements VK, Leips J, Ostrand-Rosenberg S (2006) Inflammation induces myeloid-derived suppressor cells that facilitate tumor progression. Journal of Immunology 176: 284–290. 10.4049/jimmunol.176.1.284 16365420

[25] Slansky JE, Rattis FM, Boyd LF, Fahmy T, Jaffee EM, et al (2000) Enhanced antigen-specific antitumor immunity with altered peptide ligands that stabilize the MHC-peptide-TCR complex. Immunity 13: 529–538. 11070171 10.1016/s1074-7613(00)00052-2

[26] Pamer EG, Harty JT, Bevan MJ (1991) Precise prediction of a dominant class I MHC-restricted epitope of Listeria monocytogenes. Nature 353: 852–855. 1719425 10.1038/353852a0 PMC2782917

[27] Dolcetti L, Peranzoni E, Ugel S, Marigo I, Fernandez Gomez A, et al (2010) Hierarchy of immunosuppressive strength among myeloid-derived suppressor cell subsets is determined by GM-CSF. European Journal of Immunology 40: 22–35. 19941314 10.1002/eji.200939903

[28] Abe F, Dafferner AJ, Donkor M, Westphal SN, Scholar EM, et al.. (2009) Myeloid-derived suppressor cells in mammary tumor progression in FVB Neu transgenic mice. Cancer Immunol Immunother. 10.1007/s00262-009-0719-2 PMC11030983 19449184

[29] Clark CE, Hingorani SR, Mick R, Combs C, Tuveson DA, et al (2007) Dynamics of the immune reaction to pancreatic cancer from inception to invasion. Cancer Res 67: 9518–9527. 17909062 10.1158/0008-5472.CAN-07-0175

[30] Donkor MK, Lahue E, Hoke TA, Shafer LR, Coskun U, et al (2009) Mammary tumor heterogeneity in the expansion of myeloid-derived suppressor cells. Int Immunopharmacol 9: 937–948. 19362167 10.1016/j.intimp.2009.03.021

[31] Pang G, Couch L, Batey R, Clancy R, Cripps A (1994) GM-CSF, IL-1 alpha, IL-1 beta, IL-6, IL-8, IL-10, ICAM-1 and VCAM-1 gene expression and cytokine production in human duodenal fibroblasts stimulated with lipopolysaccharide, IL-1 alpha and TNF-alpha. Clinical and experimental immunology 96: 437–443. 8004813 10.1111/j.1365-2249.1994.tb06048.x PMC1534573

[32] Fibbe WE, Daha MR, Hiemstra PS, Duinkerken N, Lurvink E, et al (1989) Interleukin 1 and poly(rI).poly(rC) induce production of granulocyte CSF, macrophage CSF, and granulocyte-macrophage CSF by human endothelial cells. Experimental hematology 17: 229–234. 2465167

[33] Suzuki A, Takahashi T, Okuno Y, Tsuyuoka R, Fukumoto M, et al (1992) IL-1 production as a regulator of G-CSF and IL-6 production in CSF-producing cell lines. British journal of cancer 65: 515–518. 1373292 10.1038/bjc.1992.106 PMC1977563

[34] Fibbe WE, van Damme J, Billiau A, Voogt PJ, Duinkerken N, et al (1986) Interleukin-1 (22-K factor) induces release of granulocyte-macrophage colony-stimulating activity from human mononuclear phagocytes. Blood 68: 1316–1321. 3535928

[35] Herrmann F, Oster W, Meuer SC, Lindemann A, Mertelsmann RH (1988) Interleukin 1 stimulates T lymphocytes to produce granulocyte-monocyte colony-stimulating factor. The Journal of clinical investigation 81: 1415–1418. 2452833 10.1172/JCI113471 PMC442572

[36] Waight JD, Hu Q, Miller A, Liu S, Abrams SI (2011) Tumor-derived G-CSF facilitates neoplastic growth through a granulocytic myeloid-derived suppressor cell-dependent mechanism. PLoS ONE 6: e27690. 22110722 10.1371/journal.pone.0027690 PMC3218014

[37] DuPre SA, Hunter KW Jr (2007) Murine mammary carcinoma 4T1 induces a leukemoid reaction with splenomegaly: association with tumor-derived growth factors. Experimental and molecular pathology 82: 12–24. 16919266 10.1016/j.yexmp.2006.06.007

[38] Xu Y, Methuku N, Coimbatore P, Fitzgerald T, Huang Y, et al (2012) Immunogenicity of an Inactivated Monovalent 2009 Influenza A (H1N1) Vaccine in Patients Who Have Cancer. The oncologist 17: 125–134. 22240540 10.1634/theoncologist.2011-0220 PMC3267811

[39] Seung LP, Rowley DA, Dubey P, Schreiber H (1995) Synergy between T-cell immunity and inhibition of paracrine stimulation causes tumor rejection. Proceedings of the National Academy of Sciences of the United States of America 92: 6254–6258. 7603979 10.1073/pnas.92.14.6254 PMC41496

[40] Sarkar D, Srivastava MK, Zhu L, Harris-White M, Kar U, et al (2012) Myeloid Suppressor Cell Depletion Augments Antitumor Activity in Lung Cancer. PLoS ONE 7: e40677. 22815789 10.1371/journal.pone.0040677 PMC3398024

[41] Serafini P, Meckel K, Kelso M, Noonan K, Califano J, et al (2006) Phosphodiesterase-5 inhibition augments endogenous antitumor immunity by reducing myeloid-derived suppressor cell function. J Exp Med 203: 2691–2702. 17101732 10.1084/jem.20061104 PMC2118163

[42] De Santo C, Serafini P, Marigo I, Dolcetti L, Bolla M, et al (2005) Nitroaspirin corrects immune dysfunction in tumor-bearing hosts and promotes tumor eradication by cancer vaccination. Proceedings of the National Academy of Sciences of the United States of America 102: 4185–4190. 15753302 10.1073/pnas.0409783102 PMC554823

[43] Kusmartsev S, Cheng F, Yu B, Nefedova Y, Sotomayor E, et al (2003) All-trans-retinoic acid eliminates immature myeloid cells from tumor-bearing mice and improves the effect of vaccination. Cancer Research 63: 4441–4449. 12907617

[44] Shi C, Hohl TM, Leiner I, Equinda MJ, Fan X, et al (2011) Ly6G+ neutrophils are dispensable for defense against systemic Listeria monocytogenes infection. Journal of Immunology 187: 5293–5298. 10.4049/jimmunol.1101721 PMC3208088 21976773

[45] Hannan R, Zhang H, Wallecha A, Singh R, Liu L, et al.. (2012) Combined immunotherapy with Listeria monocytogenes-based PSA vaccine and radiation therapy leads to a therapeutic response in a murine model of prostate cancer. Cancer immunology, immunotherapy : CII. 10.1007/s00262-012-1257-x PMC11028516 22644735

[46] Chakraborty M, Abrams SI, Coleman CN, Camphausen K, Schlom J, et al (2004) External beam radiation of tumors alters phenotype of tumor cells to render them susceptible to vaccine-mediated T-cell killing. Cancer Research 64: 4328–4337. 15205348 10.1158/0008-5472.CAN-04-0073

[47] Reits EA, Hodge JW, Herberts CA, Groothuis TA, Chakraborty M, et al (2006) Radiation modulates the peptide repertoire, enhances MHC class I expression, and induces successful antitumor immunotherapy. The Journal of experimental medicine 203: 1259–1271. 16636135 10.1084/jem.20052494 PMC3212727

[48] Kwilas AR, Donahue RN, Bernstein MB, Hodge JW (2012) In the field: exploiting the untapped potential of immunogenic modulation by radiation in combination with immunotherapy for the treatment of cancer. Frontiers in oncology 2: 104. 22973551 10.3389/fonc.2012.00104 PMC3434425

[49] Takeshima T, Chamoto K, Wakita D, Ohkuri T, Togashi Y, et al (2010) Local radiation therapy inhibits tumor growth through the generation of tumor-specific CTL: its potentiation by combination with Th1 cell therapy. Cancer Research 70: 2697–2706. 20215523 10.1158/0008-5472.CAN-09-2982

[50] Demaria S, Kawashima N, Yang AM, Devitt ML, Babb JS, et al (2005) Immune-mediated inhibition of metastases after treatment with local radiation and CTLA-4 blockade in a mouse model of breast cancer. Clin Cancer Res 11: 728–734. 15701862

[51] Gough MJ, Crittenden MR, Sarff M, Pang P, Seung SK, et al (2010) Adjuvant therapy with agonistic antibodies to CD134 (OX40) increases local control after surgical or radiation therapy of cancer in mice. J Immunother 33: 798–809. 20842057 10.1097/CJI.0b013e3181ee7095 PMC3563298

[52] Yokouchi H, Yamazaki K, Chamoto K, Kikuchi E, Shinagawa N, et al (2008) Anti-OX40 monoclonal antibody therapy in combination with radiotherapy results in therapeutic antitumor immunity to murine lung cancer. Cancer Sci 99: 361–367. 18201271 10.1111/j.1349-7006.2007.00664.x PMC11160032

[53] Shi W, Siemann DW (2006) Augmented antitumor effects of radiation therapy by 4–1BB antibody (BMS-469492) treatment. Anticancer research 26: 3445–3453. 17094465

[54] Chi CH, Wang YS, Yang CH, Chi KH (2010) Neoadjuvant immunotherapy enhances radiosensitivity through natural killer cell activation. Cancer biotherapy & radiopharmaceuticals 25: 39–45. 20187795 10.1089/cbr.2009.0699

